# Molecular pathways in cardiovascular disease under hypoxia: Mechanisms, biomarkers, and therapeutic targets

**DOI:** 10.7555/JBR.38.20240387

**Published:** 2025-03-20

**Authors:** Izzatullo Abdullaev, Ulugbek Gayibov, Sirojiddin Omonturdiev, Sobirova Fotima, Sabina Gayibova, Takhir Aripov

**Affiliations:** 1 A. S. Sadykov Institute of Bioorganic Chemistry, Science Academy of Uzbekistan, Laboratory of Plant CytoProtectors, Tashkent 100007, Uzbekistan; 2 Alfrganus University, Faculty of Medicine, Department of Pharmacy and Chemistry, Tashkent 100190, Uzbekistan

**Keywords:** oxidative stress, mitoK_ATP_, mPTP, HIF-1α, mitochondrion, heart ischemia

## Abstract

Chronic hypoxia is a key factor in the pathogenesis of cardiovascular diseases, including ischemia, heart failure, and hypertension. Under hypoxia, oxygen deficiency disrupts oxidative phosphorylation in mitochondria, impairing ATP production and generating reactive oxygen species (ROS). These reactive species induce mitochondrial dysfunction, leading to oxidative stress, calcium imbalance, and activation of apoptosis pathways. The mitochondrial ATP-sensitive potassium channel (mitoK_ATP_) and mitochondrial permeability transition pore (mPTP) channels are particularly affected, contributing to membrane potential loss, cytochrome c release, and cell death. This review delves into the molecular mechanisms underlying hypoxia-induced cardiovascular diseases, with a focus on mitochondrial impairment, ion channel dysfunction, and ROS overproduction. Additionally, we examine hypoxia-inducible factor 1-alpha (HIF-1α) as a biomarker of cellular adaptation and discuss therapeutic strategies targeting mitochondrial function and oxidative stress. Antioxidants and compounds modulating key ion channels, such as mitoK_ATP_ and mPTP, are highlighted as promising interventions for mitigating hypoxia-induced damage. Furthermore, we emphasize the potential of integrating *in vitro*, *in vivo*, and *in silico* studies to develop novel therapies aimed at preserving mitochondrial integrity and preventing cardiovascular diseases.

## Introduction

Cardiovascular diseases remain a leading cause of morbidity and mortality worldwide, with hypoxia and ischemia serving as key pathological drivers of many of these conditions. Hypoxia, a state of reduced oxygen availability, disrupts cellular energy homeostasis and triggers a cascade of molecular events that compromise cardiac function. Understanding the molecular pathways underlying hypoxia-induced cardiovascular dysfunction is essential for identifying biomarkers and developing targeted therapeutic interventions^[1]^.

Mitochondria, membrane-bound organelles found in nearly all eukaryotic cells, play a pivotal role in energy production and cellular homeostasis, making them central to the pathophysiology of hypoxia-induced cardiovascular diseases. These organelles are critical for generating ATP through oxidative phosphorylation and regulating biological processes such as calcium signaling, cell growth, differentiation, apoptosis, and the cell cycle. Structurally, the outer and inner membranes of mitochondria compartmentalize the intermembrane space and matrix^[2]^. Their dynamic processes, including fission, fusion, biogenesis, and autophagy, ensure that mitochondrial populations remain functional and adapt to cellular demands.

Under hypoxic and ischemic conditions, mitochondria are particularly vulnerable because of their dependence on oxygen for ATP production. Oxygen deprivation disrupts oxidative phosphorylation, impairing ATP synthesis and leading to bioenergetic failure^[3]^. This dysfunction is particularly critical in tissues with high energy demands, such as the heart. During hypoxia, mitochondrial calcium homeostasis is also often dysregulated, resulting in excessive calcium uptake and the opening of the mitochondrial permeability transition pore (mPTP). This process triggers necrotic or apoptotic cell death, exacerbating tissue damage in conditions such as heart failure and ischemic injury.

Additionally, mitochondrial dysfunction is significantly associated with increased oxidative stress^[4]^. Reactive oxygen species (ROS) generated during hypoxia contribute to lipid peroxidation, protein damage, and mitochondrial DNA mutations, further impairing mitochondrial function. These effects are amplified during ischemic episodes, where prolonged oxygen deprivation leads to calcium overload, oxidative damage, and cell death, accelerating the progression of ischemic heart disease.

Mitochondrial biogenesis, which requires the coordinated expression of nuclear and mitochondrial genes, is another crucial process disrupted under hypoxia^[5]^. Defects in mitochondrial biogenesis or mutations in mitochondrial DNA may result in inherited disorders, particularly in tissues reliant on oxidative phosphorylation, such as the heart. Understanding how hypoxia affects mitochondrial dynamics and function is essential for developing strategies to mitigate ischemic damage^[6]^.

This review focuses on the molecular pathways linking hypoxia and cardiovascular disease, with a focus on the roles of mitochondria. Unraveling the mechanisms underlying hypoxia-induced cardiac dysfunction may identify potential therapeutic targets and biomarkers to improve clinical outcomes in ischemic cardiovascular diseases.

## Pathophysiological mechanisms

Biological macromolecules, namely proteins, carbohydrates, and fats, are essential for sustaining energy production in the human body. Among these, carbohydrates play a particularly prominent role because of their capacity to generate substantial energy through metabolic pathways. As the primary fuel source for cells, carbohydrates are especially vital in tissues with high energy demands, such as muscle and nerve cells, where rapid and efficient ATP production is critical for maintaining function^[7]^.

The central role of carbohydrates in metabolism is evident in their involvement in key bioenergetic pathways, including glycolysis, the citric acid cycle, and oxidative phosphorylation^[8]^. These processes collectively convert glucose and other sugars into ATP, the cell's primary energy currency. Efficient carbohydrate metabolism ensures sufficient energy to support cellular activities, such as muscle contraction, ion transport, and biosynthesis^[9]^.

Disruptions in metabolic homeostasis have significant consequences for energy production. When the delicate balance of carbohydrate metabolism is disturbed because of genetic factors, diseases such as diabetes, or environmental stressors, the breakdown and utilization of glucose may become inefficient^[10]^. This inefficiency leads to the accumulation of metabolic intermediates or end products that are not fully processed, thereby limiting ATP production. Consequently, reduced ATP levels impair various cellular functions, potentially leading to cellular dysfunction, fatigue, and metabolic disorders^[11]^.

Diminished ATP production is particularly detrimental in high-energy demand organs, such as the heart, where a consistent and efficient energy supply is essential for normal function. Inadequate energy production may lead to cellular stress and contribute to diseases, such as ischemic heart disease, in which oxygen and nutrient supply are already compromised, exacerbating energy deficits and cellular damage^[12]^. Thus, understanding the critical role of carbohydrates in energy metabolism is key to elucidating the mechanisms underlying metabolic and cardiovascular diseases^[13]^.

### Bridge between hypoxia and ischemic cardiovascular disease: The role of mitochondria

Mitochondria play a pivotal role in linking hypoxia to ischemia-induced cardiovascular dysfunction. Under conditions of oxygen deprivation, the impaired capacity of mitochondria to perform oxidative phosphorylation triggers a cascade of metabolic and structural disruptions, which are key drivers of ischemic pathology^[14]^.

#### Disruption of oxidative phosphorylation and ATP production

Hypoxia inhibits the electron transport chain (ETC), halting oxidative phosphorylation and drastically reducing ATP synthesis^[15]^. This energy deficit profoundly affects cellular function, particularly in energy-demanding tissues such as the heart. The failure to produce sufficient ATP compromises the activity of ATP-dependent ion channels, including mitochondrial ATP-sensitive potassium (mitoK_ATP_) channels, which are essential for maintaining the mitochondrial membrane potential (Δψm) and cellular homeostasis^[16]^.

#### mitoK_ATP_ channel dysfunction

The mitoK_ATP_ channel plays a key role in regulating the Δψm by responding to fluctuations in cellular energy levels. During hypoxia, ATP depletion renders these channels non-functional, leading to mitochondrial depolarization. This depolarization disrupts ion homeostasis and triggers a cascade of events that impair other ion channels, ultimately compromising the ability of cardiac and vascular cells to maintain their physiological functions^[17–18]^.

#### ROS and lipid peroxidation

Hypoxia-induced mitochondrial dysfunction results in excessive ROS production, which leads to lipid peroxidation in the mitochondrial lipid bilayer, compromising the integrity of mitochondrial membranes and releasing pro-apoptotic factors and other mitochondrial contents into the cytosol. ROS-mediated damage extends to other organelles, with the sarcoplasmic reticulum being a primary target^[19]^. Under hypoxic conditions, impaired mitochondrial oxidative phosphorylation amplifies ROS production, which acts as a signaling mechanism to promote vascular remodeling. Additionally, hypoxia stabilizes hypoxia-inducible factor 1-alpha (HIF-1α), a key regulator of the cellular response to oxygen deprivation. HIF-1α activation drives the expression of genes involved in glycolysis, angiogenesis, and cell proliferation, contributing to the maladaptive vascular remodeling characteristic of pulmonary hypertension^[20]^.

#### Sarcoplasmic reticulum and calcium dysregulation

ROS damages the lipid bilayer and protein structures of the sarcoplasmic reticulum, impairing the function of its calcium channels. Critical amino acids involved in channel gating are modified or degraded, disrupting calcium homeostasis. This dysfunction contributes to impaired cardiac contractility and vascular tone regulation, both hallmark features of ischemia^[21]^.

#### Membrane potential and ion channel dysfunction

The loss of ATP and subsequent ATP-sensitive potassium (K_ATP_) channel dysfunction disrupts the membrane potential of smooth muscle cells, affecting all ion channels in the vascular wall, including those regulating intracellular calcium levels, sodium influx, and potassium efflux. The failure of these channels exacerbates vascular dysfunction, leading to vasoconstriction, increased vascular resistance, and impaired blood flow, which are key contributors to ischemic injury^[22]^.

#### Systemic effect of ischemia

The interplay between mitochondrial dysfunction, ROS production, and ion channel failure creates a vicious cycle of cellular damage. Under ischemic conditions, these events contribute to endothelial dysfunction, vascular remodeling, and cardiomyocyte death, ultimately leading to tissue necrosis and organ dysfunction.

## Mitochondrial bioenergetics

**Figure 1:**
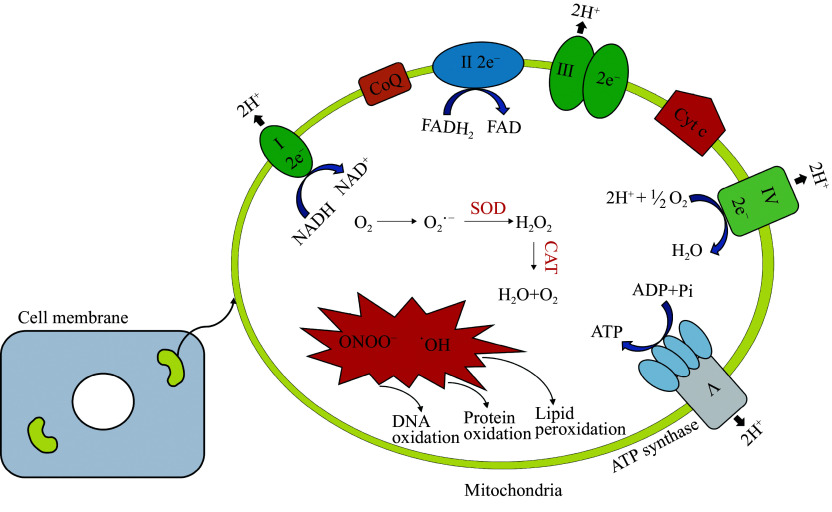
Generation of reactive oxygen species (ROS) in mitochondria. The mitochondrial electron transport chain consists of five multi-subunit enzyme complexes embedded in the inner mitochondrial membrane. This figure illustrates the flow of electrons through complexes Ⅰ–Ⅳ and the associated sites of superoxide anion (O_2_^•−^) generation as by-products of incomplete oxygen reduction. Abbreviations: SOD, superoxide dismutase; CAT, catalase; Cyt c, cytochrome c.

Mitochondria serve as the central hub for energy production, completing the oxidation of sugars, fats, and proteins to generate ATP. These molecules are broken down into acetyl-CoA, which enters the citric acid (Krebs) cycle in the mitochondrial matrix (***Fig. 1***). Sugars, after undergoing glycolysis in the cytosol, enter the mitochondria as pyruvate, which is converted to acetyl-CoA by pyruvate dehydrogenase^[23]^. Fatty acids undergo beta-oxidation to produce acetyl-CoA, while certain amino acids are converted into pyruvate, acetyl-CoA, or citric acid cycle intermediates by specific enzymes. In the citric acid cycle, acetyl-CoA donates its two-carbon group to oxaloacetate, forming citrate. This citrate is progressively oxidized back to oxaloacetate, releasing two molecules of carbon dioxide and transferring electrons to NADH and FADH_2_^[24]^. These electron carriers then deliver electrons to the ETC in the mitochondrial inner membrane. The ETC, composed of multiple protein complexes, uses these electrons to pump protons across the mitochondrial membrane, generating a proton gradient. This gradient drives ATP synthesis *via* oxidative phosphorylation, where ATP synthase (complex Ⅴ) produces ATP by harnessing the energy from protons moving down their gradient. Complex Ⅰ (NADH dehydrogenase) receives electrons from NADH, while complex Ⅱ (succinate dehydrogenase) accepts electrons from FADH_2_^[25]^. Coenzyme Q transfers these electrons to complex Ⅲ, which then passes them to cytochrome c. Finally, complex Ⅳ reduces oxygen to water using these electrons, while protons continue to be pumped across the membrane, further strengthening the proton gradient. ATP synthase functions as a molecular motor, synthesizing ATP from ADP and phosphate as protons flow through it^[26]^. Each full rotation of the ATP synthase produces three ATP molecules, though the exact number of protons required for each ATP molecule may vary because of proton leakage. NADH typically generates three ATP molecules, while FADH_2_ produces two ATP molecules, as complex Ⅱ does not pump protons. However, proton leakage and electron slippage from the ETC may lead to superoxide formation, a ROS. This superoxide contributes to oxidative stress, which is implicated in numerous diseases and aging. Oxidative stress occurs when excess ROS damages cellular components, potentially leading to conditions such as atherosclerosis, diabetes, neurodegenerative disorders, and cancer^[27]^.

### The role of mitoK_ATP_ and mPTP ion channels in regulating mitochondrial membrane

#### mitoK_ATP_

The mitoK_ATP _channel plays a pivotal role in maintaining the Δψm by facilitating K^+^ influx into the mitochondrial inner membrane^[28]^. This channel regulates several critical processes, including the activity of other ion channels, particularly during cellular stress or periods of high energy demand. Its function is highly dependent on ATP availability^[29]^, rendering it one of the first to be affected when ATP production declines. The mitoK_ATP_ disruptions induced by insufficient energy may lead to an imbalance in mitochondrial K^+^ homeostasis, destabilize the Δψm, and may also have downstream consequences, notably triggering the opening of the mPTP^[30]^.

The opening of the mPTP may lead to a collapse of the Δψm and mitochondrial dysfunction. The Δψm collapse may trigger the release of pro-apoptotic factors, increase ROS production, and disrupt calcium homeostasis, eventually driving the cell toward necrosis or apoptosis^[31]^. In cardiovascular diseases, particularly under hypoxic conditions like ischemia, mitoK_ATP_ channels play a protective role by helping to maintain the Δψm. If mitoK_ATP_ function is compromised because of low ATP levels, the heart becomes more vulnerable to ischemic damage, as its defense against oxidative stress and calcium overload is weakened^[32]^.

Therefore, mitoK_ATP_ channels are considered critical therapeutic targets for preventing ischemia-reperfusion injury. Their regulation may preserve mitochondrial function, delay mPTP opening, and maintain cellular viability during hypoxic stress. This underscores their essential role in energy metabolism, mitochondrial dynamics, and disease progression, especially in hypoxia-induced cardiovascular conditions such as myocardial ischemia^[33]^.

Various scientific studies have highlighted the crucial role of mitoK_ATP_ in adapting to low oxygen conditions (*i.e.*, hypoxia). These channels, located in the inner membrane of mitochondria, are considered potential targets for drugs with anti-hypoxic effects. Investigators have isolated a protein with properties consistent with this channel, which is inhibited by physiological ATP levels. As a result, it has been termed the mitochondrial ATP-inhibited potassium channel, also known as mitoK_ATP_^[34]^.

The biophysical characteristics and physiological functions of mitoK_ATP_ channels are well understood. They play a key role in resisting oxygen deprivation, particularly in protecting the heart muscle (myocardium) during ischemia, a condition where blood flow and oxygen supply to the heart are restricted. Several synthetic mitoK_ATP_ activators have been identified, many of which show promise as cardioprotective agents. A natural activator of these channels is uridine-5′-diphosphate (UDP), while synthetic activators include diazoxide and nicorandil, which also activate potassium channels in the cell membrane at higher concentrations. Additionally, certain hormones, such as β-estradiol and testosterone, are known to activate mitoK_ATP_ channels^[35]^.

Studies have shown that diphosphate nucleotides, including ADP and GDP, can activate mitoK_ATP_ channels, with UDP exerting the most significant effect. Precursors to UDP, such as uridine and uridine monophosphate, have been proposed as potential treatments for preventing hypoxia. In a rat model of myocardial infarction, these substances reduced infarct size (damaged area), restored ATP and creatine phosphate levels, enhanced antioxidant defenses, reduced ROS production, and stabilized heart rhythms. However, these beneficial effects were abolished when mitoKATP channels were blocked by inhibitors like glibenclamide, confirming their critical role in heart protection during ischemia^[36]^.

### mPTP

The mPTP is a promising pharmacological target for regulating cell adaptation to hypoxia. This protein complex forms a channel that spans both the outer and inner mitochondrial membranes and functions through conformational changes in its protein components, thereby regulating metabolic activity^[37]^.

The structural components of the mPTP include the voltage-dependent anion channel and the peripheral benzodiazepine receptor in the outer mitochondrial membrane. In the inner membrane, the adenine nucleotide translocase is located near cyclophilin D in the mitochondrial matrix. The mPTP opening occurs under pathological conditions such as stroke, traumatic brain injury, neurodegenerative diseases, hepatic encephalopathy, muscular dystrophy, and myocardial infarction. In myocardial ischemia, the mPTP, which remains closed during ischemia, plays a key role in reperfusion injury by opening upon the restoration of blood flow and contributing to cellular damage^[38]^.

Beyond its structural and metabolic functions, the mPTP plays a regulatory role in mitochondrial apoptosis. While mPTP formation and opening are not the sole mechanisms for releasing mitochondrial intermembrane proteins into the cytoplasm, the extent and duration of its opening determine cell fate following events like a stroke. A minor permeability increase may allow cell recovery, whereas significant mPTP opening can lead to apoptosis. mPTP opening facilitates the influx of water and ions into the mitochondrial matrix, resulting in mitochondrial swelling that damages the outer membrane and triggers the release of apoptotic proteins, including apoptosis-inducing factors, secondary mitochondrial activators of caspases, and certain procaspases. Additionally, mPTP opening releases cytochrome c, a key ETC component that binds to apoptotic protease-activating factor 1 (Apaf-1) in the cytoplasm, forming the apoptosome and initiating a cascade that activates caspase-9, -3, and -7, ultimately causing apoptosis^[39]^.

Several endogenous factors induce mPTP opening, including Ca^2+^, adenine nucleotides, nitric oxide, ROS, pyrimidine and thiol redox states, B-cellleukemia/lymphoma 2 (BCL-2) family proteins, excitatory amino acids, and certain fatty acids.

Cyclophilin D inhibitors, such as cyclosporin A and its analogs, block the mPTP, making it a potential therapeutic target. Selective mPTP inhibitors may offer effective treatment for ischemic heart disease, ischemic brain conditions, and neurodegenerative diseases like Alzheimer's and Huntington's disease. Additionally, modifying the mPTP protein conformation can influence cell survival and human longevity. Given its role in programmed cell death, the mPTP is also a potential target for anticancer therapies designed to induce apoptosis in proliferating cancer cells^[40]^.

Ubiquinone (coenzyme Q10, CoQ10) has been demonstrated to inhibit mPTP opening in the myocardium during ischemia-reperfusion. During ischemia and subsequent reperfusion, myocardial cells undergo apoptosis; however, CoQ10 exerts a protective effect by attenuating calcium-induced mitochondrial swelling, especially when the respiratory chain is suppressed. Investigators have proposed that ubiquinone-binding sites, regulated by the mitochondrial respiratory chain, exist within the pore structure itself. The protective effect of CoQ10 may also stem from its ability to restructure proteins within the mPTP complex. Therefore, ubiquinone functions not only as a cofactor in the respiratory chain but also as an inhibitor of mPTP, contributing to its anti-hypoxic effect^[41]^.

## Hypoxia and cellular adaptations

Hypoxia occurs when there is an insufficient oxygen supply to tissues, which may result from respiratory diseases, disruptions in iron metabolism that reduce iron availability, or impaired erythropoiesis that decreases the number of red blood cells. Collectively, these processes affect the body's ability to deliver adequate oxygen. The term "hypoxia" is derived from "hypo" (low) and "oxys" (oxygen), meaning a deficiency of oxygen. At the cellular level, oxygen is essential for the breakdown of biological molecules, and its shortage can have severe consequences. For instance, in diseases such as COVID-19, where oxygen transport is compromised, cells fail to meet their oxygen demand^[42]^.

**Figure 2:**
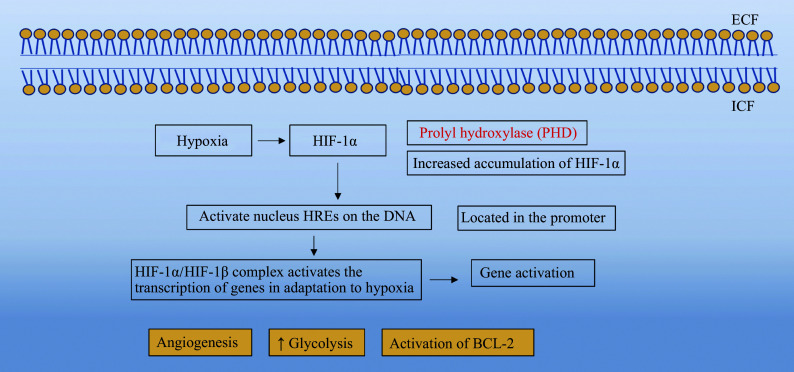
Overview of the HIF pathway under normoxia and hypoxia. Under normal oxygen levels, HIF-α is hydroxylated by prolyl hydroxylase (PHD), leading to its degradation *via* the von Hippel-Lindau protein-ubiquitin-proteasome pathway. During hypoxia, PHD activity is inhibited, allowing HIF-α to accumulate, enter the nucleus, dimerize with HIF-1β, and activate target genes involved in adaptation to low oxygen. Abbreviations: HIF-1α, hypoxia-inducible factor 1-alpha; HREs, hypoxia-responsive elements; ECF, extracellular fluid; ICF, intracellular fluid.

Under normoxia, HIF-1α undergoes prolyl hydroxylase (PHD)-mediated proteasomal degradation. Under hypoxia, this degradation is inhibited, leading to an increase in HIF-1α levels. As HIF-1α accumulates in the cytoplasm, it migrates to the nucleus, where it binds to hypoxia-inducible factor 1-beta (HIF-1β) at specific DNA promoter regions (***Fig. 2***). The HIF-1α/HIF-1β complex activates the expression of a series of proteins responsible for cellular adaptation to hypoxia^[43]^, such as BCL-2, which regulates proliferation and apoptosis; LDHA, which promotes anaerobic metabolism; PDK-1, which inhibits the TCA cycle; GLUT1, which facilitates increased glucose uptake to enhance anaerobic metabolism; endothelial nitric oxide synthase (eNOS), which modulates vascular tone; ANGPT, which promotes angiogenesis; TF, which regulates iron metabolism; EPO, which stimulates erythropoiesis (red blood cell production); CD18, which is involved in inflammation; and TIMP metallopeptidase inhibitor 1 (TIMP-1), which aids in maintaining matrix and barrier functions.

**Figure 3:**
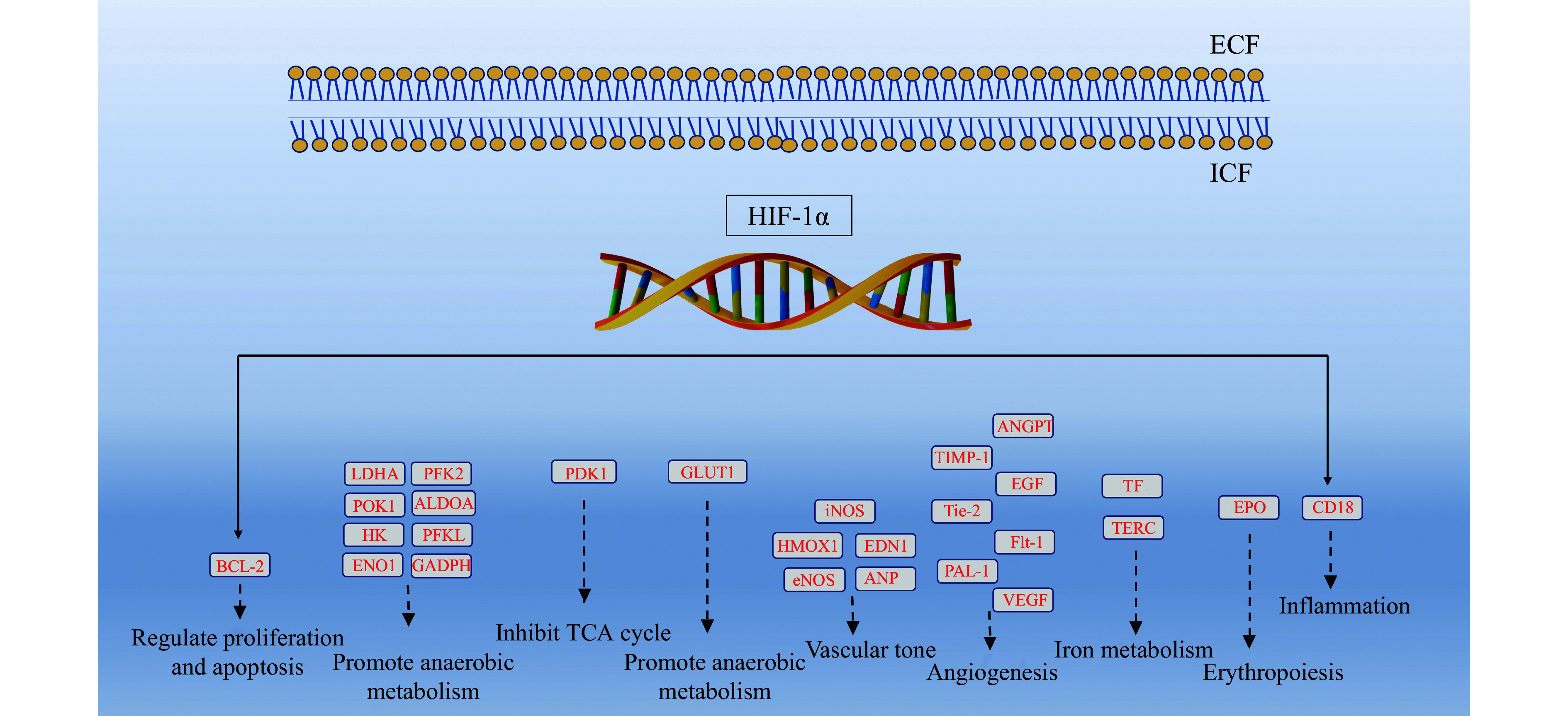
Schematic representation of the hypoxia-inducible factor 1-alpha (HIF-1α) signaling pathway. The figure illustrates the activation of hypoxia-responsive genes mediated by stabilized HIF-1α under low oxygen conditions, highlighting key molecular interactions and downstream targets. Abbreviations: ALDOA, aldolase A; ANGPT, angiopoietin; ANP, atrial natriuretic peptide; BCL-2, B-cell leukemia/lymphoma 2; CD18, cluster of differentiation 18; ECF, extracellular fluid; EPO, erythropoietin; ENO1, enolase 1; EGF, epidermal growth factor; Flt-1, fms-like tyrosine kinase 1; GAPDH, glyceraldehyde-3-phosphate dehydrogenase; GLUT1, glucose transporter 1; HMOX1, heme oxygenase 1; HK, hexokinase; ICF, intracellular fluid; iNOS, inducible nitric oxide synthase; LDHA, lactate dehydrogenase A; PFK2, phosphofructokinase 2; PDK1, pyruvate dehydrogenase kinase isozyme 1; POK1, phragmoplast orienting kinesin 1; PFKL, ATP-dependent 6-phosphofructokinase, liver type; TIMP-1, tissue inhibitor metalloproteinase-1; TERC, telomerase RNA component; TF, tissue factor; VEGF, vascular endothelial growth factor.

These proteins help cells adapt to the reduced oxygen environments by shifting metabolism from aerobic (oxygen-dependent) to anaerobic (oxygen-independent) pathways (***Fig. 3***). For example, GLUT1 increases glucose transport into cells, ensuring a higher availability of glucose for glycolysis, an anaerobic process that breaks down glucose into lactic acid, generating a small amount of ATP (two molecules). Under normal oxygen conditions, the TCA cycle and oxidative phosphorylation produce significantly more ATP. However, in hypoxia, PDK1 inhibits the TCA cycle to prevent electron buildup, which would otherwise lead to the generation of ROS, such as hydrogen peroxide (H_2_O_2_) and superoxide anion (O_2_^•−^), that are harmful to cells^[44]^.

The shift in metabolic pathways and the activation of protective genes are critical for cell survival during hypoxic stress. Without these adaptations, prolonged hypoxia may lead to cell death through mechanisms such as apoptosis or necrosis. Therefore, understanding the molecular responses to hypoxia offers insight into potential therapeutic targets for conditions where oxygen deficiency plays a significant role, such as ischemia, stroke, or chronic lung disease.

### Chronic hypoxia and mitochondrial dysfunction

Chronic hypoxia, or prolonged oxygen deficiency, presents significant challenges for cellular adaptation that the body cannot endure indefinitely. Although cells can make short-term adjustments to cope with hypoxia, prolonged oxygen deprivation leads to severe disruptions in key metabolic processes, particularly oxidative phosphorylation^[45]^.

Oxygen plays a critical role in the ETC in mitochondria, especially at complex Ⅳ (cytochrome c oxidase), where the final transfer of electrons to oxygen produces water. Under chronic hypoxia, oxygen scarcity inhibits complex Ⅳ activity, halting ATP synthase activity and reducing ATP production. This ATP deficiency initially affects mitoK_ATP_ channels, which help maintain the Δψm. Dysfunctional mitoK_ATP_ channels disrupt the delicate balance of the membrane potential, inducing the opening of other ion channels, such as the mPTP^[46]^.

The malfunction of complex Ⅳ also results in an accumulation of electrons and protons within complexes Ⅰ and Ⅲ from the Krebs cycle. These unused electrons contribute to the generation of ROS, such as H_2_O_2_ and O_2_^•−^. Excessive ROS production exacerbates mitochondrial damage, leading to oxidative stress, which in turn further disrupts cellular functions^[47]^.

**Figure 4:**
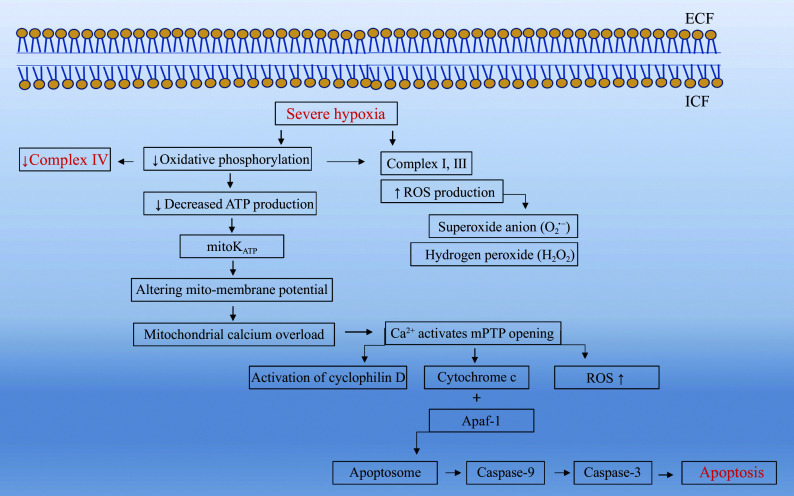
Effects of chronic hypoxia on mitochondrial function. The figure depicts how severe hypoxia triggers mitochondrial dysfunction, including excessive reactive oxygen species (ROS) production, disruption of ion channel activity, and activation of apoptotic pathways. Abbreviations: ECF, extracellular fluid; ICF, intracellular fluid; mPTP, mitochondrial permeability transition pore; mitoK_ATP_, mitochondrial ATP-sensitive potassium channel.

When ATP levels become insufficient to sustain normal cellular functions, mitoK_ATP_ channels lose their regulatory capacity, triggering Ca^2+^ influx into the mitochondrial matrix (***Fig. 4***). The mPTP channel normally helps regulate calcium homeostasis within mitochondria. However, an excessive increase in Ca^2+^ influx triggers mPTP channel opening, which leads to mitochondrial depolarization, loss of membrane integrity, and the release of pro-apoptotic factors, including cytochrome c, into the cytosol^[48]^.

Cytochrome c release is a hallmark event in the intrinsic pathway of apoptosis. Once in the cytosol, cytochrome c binds to apoptotic protease activating factor 1 (APAF-1), forming the apoptosome complex. This complex activates caspases, starting with caspase-9, which then activates caspase-3, leading to the execution phase of apoptosis, or programmed cell death. At this stage, the cell undergoes irreversible damage. Although apoptosis ensures that damaged or malfunctioning cells do not survive under chronic hypoxic conditions, it also results in the loss of functional tissues, contributing to the progression of diseases associated with chronic hypoxia, such as chronic obstructive pulmonary disease, heart failure, and ischemic injury^[49]^.

### Mechanisms and implications for cardiovascular disease

Chronic hypoxia-induced mitochondrial dysfunction highlights the delicate balance of cellular energy systems and the critical role of oxygen. As mitochondria are the primary energy producers in cells, their impairment under hypoxic conditions triggers a cascade of cellular failures. The body's inability to produce sufficient ATP affects not only energy-demanding processes but also the regulation of oxidative stress. Although the shift to anaerobic metabolism initially serves as a compensatory mechanism, it ultimately becomes insufficient to sustain cellular functions in the long term.

Additionally, the accumulation of ROS during prolonged hypoxia further accelerates cellular damage. ROS not only harm mitochondrial components but also initiate lipid peroxidation, protein oxidation, and DNA damage, potentially leading to mutations and cell death if not properly countered by antioxidant defenses^[50]^.

Understanding the cellular mechanisms underlying chronic hypoxia provides valuable insights into potential therapeutic strategies. Targeting ROS generation, supporting mitochondrial functions, and enhancing the cell's antioxidant capacity are all promising approaches for mitigating the detrimental effects of chronic hypoxia.

**Figure 5:**
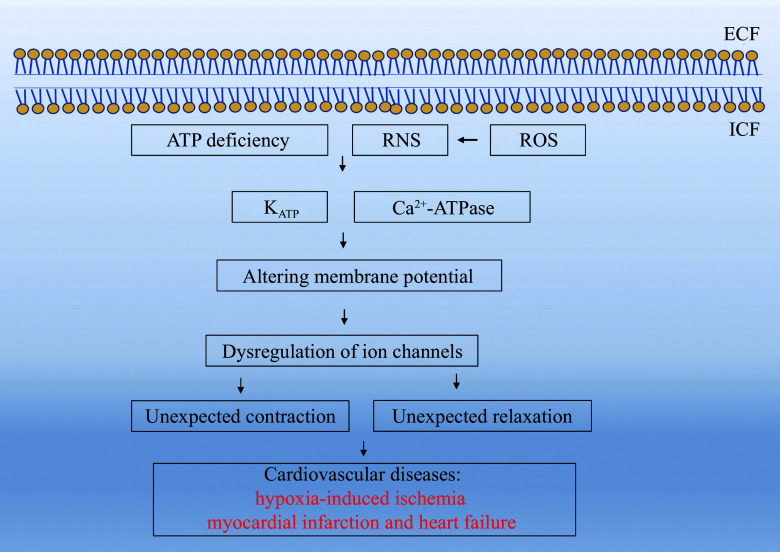
ATP depletion-driven disturbance of ion channel function in cardiovascular pathology. This diagram depicts how ATP deficiency, together with reactive oxygen/nitrogen species (ROS/RNS), affects K_ATP_ and Ca^2+^-ATPase activity, alters membrane potential, and disrupts ion channel regulation. These changes result in abnormal contraction or relaxation, contributing to cardiovascular diseases such as hypoxia-induced ischemia, myocardial infarction, and heart failure. Abbreviations: ECF, extracellular fluid; ICF, intracellular fluid.

Chronic oxygen deficiency (hypoxia) disrupts oxidative phosphorylation, a key process in cellular energy production (***Fig. 5***). As a result, electrons that normally transfer to complex Ⅳ in the ETC would instead accumulate in complexes Ⅰ and Ⅲ and interact with molecular oxygen to form ROS, including H_2_O_2_ and O_2_^•−^. ROS accumulation, combined with ATP deficiency, plays a critical role in the development of cardiovascular diseases^[51]^.

ROS are highly reactive and primarily target the mitochondrial lipid membrane, leading to lipid peroxidation and compromising membrane integrity. As ROS levels increase, they diffuse into the cytosol and interact with eNOS in cardiovascular cells, leading to the formation of reactive nitrogen species (RNS), including peroxynitrite (ONOO^−^), a potent oxidizing agent (***Fig. 5***). Together, ROS and RNS damage cellular components, particularly lipid-rich organelles^[52]^.

**Figure 6:**
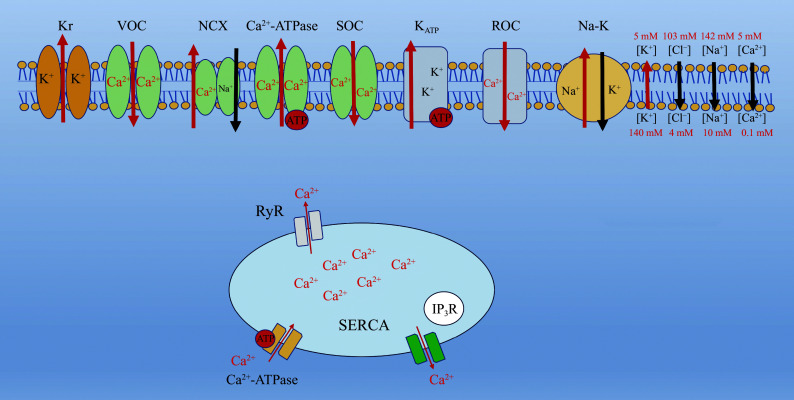
Smooth muscle ion channels. Voltage-gated calcium channels facilitate calcium influx, triggering contraction. Potassium channels include ATP-sensitive potassium channels (K_ATP_) and large-conductance calcium-activated potassium channels, which regulate membrane potential and relaxation. Voltage-gated sodium channels contribute to depolarization. Calcium-activated chloride channels play a role in depolarization and contraction. The sarcoplasmic reticulum releases and uptakes calcium through RyRs and SERCA pumps, modulating cytosolic calcium levels. Abbreviations: Kr, potassium channels; VOC, voltage-operated channels; NCX, sodium-calcium exchanger; SOC, stock-operated channels; ROC, receptor-operated channels; RyR, ryanodine receptor; IP_3_R, inositol-3-phosphate; SERCA, sarcoplasmic reticulum calcium channels.

One of the primary targets of this oxidative damage is the sarcoplasmic reticulum, an organelle critical for calcium storage and release in cardiac muscle cells. The sarcoplasmic reticulum relies on specific ion channels to regulate Ca^2+^ concentrations, which are essential for muscle contraction and relaxation. ROS and RNS modify amino acid residues (*e.g.*, dityrosine and tryptophan) in these ion channels, impairing their functions (***Fig. 6***). This disruption impairs the normal cycling of Ca^2+^, leading to altered intracellular calcium homeostasis, a crucial factor in heart function^[53]^.

K_ATP_ channels in cardiac cells rely on sufficient ATP to function correctly. In the absence of ATP, these channels become dysfunctional, disturbing the resting membrane potential of the cells. This change in membrane potential may activate other voltage-gated ion channels, leading to abnormal electrical activity, such as hyperpolarization or depolarization of the cell membrane. Consequently, calcium flux becomes imbalanced, potentially leading to either an overload of Ca^2+^ or a significant reduction in its availability^[54]^. Such calcium imbalance can trigger unpredictable contractions or relaxations in blood vessels, contributing to various cardiovascular diseases, including hypertension, ischemic stroke, and myocardial infarction. If not properly managed, these conditions can be life-threatening.

### The role of ROS and RNS in oxidative damage and cardiovascular pathophysiology

The role of ROS and RNS in cardiovascular diseases extends beyond simple oxidative damage. Both ROS and RNS can interfere with signaling pathways that regulate vascular tone, inflammation, and apoptosis. In the context of chronic hypoxia, elevated ROS levels activate inflammatory cascades, leading to the upregulation of pro-inflammatory cytokines that exacerbate tissue damage. Additionally, ROS can trigger apoptotic pathways, leading to programmed cell death, which contributes to the loss of functional cardiac cells and the progression of heart failure.

Endothelial cells, which line blood vessels, are particularly sensitive to oxidative damage. ROS and RNS may impair nitric oxide (NO) signaling, which is essential for vasodilation and maintaining proper blood flow. When NO production is disrupted, blood vessels constrict, increasing vascular resistance and contributing to conditions such as hypertension and atherosclerosis^[55]^.

Additionally, mitochondrial dysfunction caused by ROS and RNS alters the energy supply to the heart, which is particularly detrimental in conditions like ischemia, where the heart already struggles to receive adequate oxygen. Impaired energy production, coupled with oxidative damage, creates a vicious cycle of cellular injury and heart failure. Understanding these pathways opens opportunities for therapeutic interventions aimed at reducing ROS production, enhancing antioxidant defenses, and protecting mitochondrial functions to prevent cardiovascular diseases.

## Research and treatment strategies for hypoxia-induced cardiovascular dysfunction

The prevention and treatment of conditions affecting mitochondrial functions, such as hypoxia, diabetes, and related cardiovascular conditions, have become critical areas of focus in modern medicine. Cardiovascular diseases, particularly those caused by chronic hypoxia, remain a leading cause of death worldwide. Therefore, developing biologically active medicinal compounds and synthetic drugs is essential for addressing these conditions.

Antioxidants have been explored to mitigate oxidative stress and prevent hypoxia-induced damage^[56]^. Experimental studies aimed at developing these treatments generally fall into three categories: *in vitro*, *in vivo*, and *in silico* experiments. Each approach provides unique insights into the underlying mechanisms and potential interventions for oxidative stress-related conditions.

### *In vitro* studies

Mitochondrial isolates and aortic tissues are commonly used in *in vitro* experiments.

**Figure 7:**
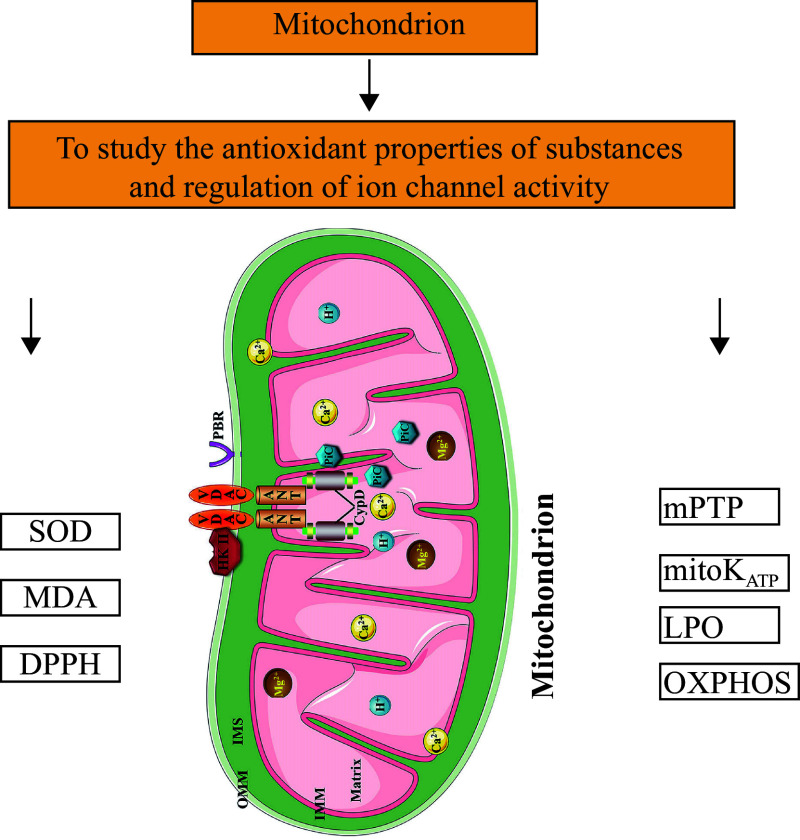
Schematic representation of experimental approaches used to evaluate mitochondrial function and antioxidant activity. This figure illustrates methods for assessing mitochondrial parameters such as mitochondrial permeability transition pore (mPTP) opening, mitochondrial ATP-sensitive potassium channel (mito_KATP_) channel activity, lipid peroxidation (LPO), and oxidative phosphorylation (OXPHOS), alongside antioxidant activity assays including superoxide dismutase (SOD), malondialdehyde (MDA), and 2,2-dipheny-1-picrylhydrazyl (DPPH), within the context of ion channel regulation and oxidative stress in mitochondria.

Mitochondrial analyses primarily focus on evaluating the antioxidant properties of compounds through four key assays (***Fig. 7***): (1) superoxide dismutase (SOD) activity assay, which measures the enzyme's ability to neutralize superoxide radicals, a major type of ROS; (2) malondialdehyde (MDA) assay, which quantifies lipid peroxidation as an indicator of oxidative damage to cell membranes; (3) 2,2-diphenyl-1-picrylhydrazyl (DPPH) assay, which assesses the free radical-scavenging activity of antioxidants; and (4) ferric reducing antioxidant power (FRAP) assay, which evaluates the reducing power of antioxidants in biological samples. These assays are typically performed using a spectrophotometer to measure oxidative stress markers and antioxidant activity. Additionally, the regulation of mitochondrial ion channels, such as mPTP and mitoK_ATP_ channels, is examined, as these channels play crucial roles in maintaining the Δψm and protecting against oxidative stress. The effects of antioxidants on oxidative phosphorylation have also been assessed using specialized techniques to measure oxygen consumption, which reflects the efficiency of mitochondrial respiration^[57]^.

**Figure 8:**
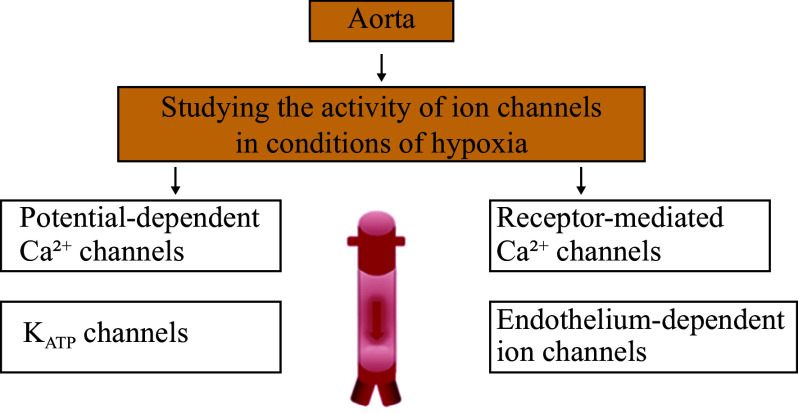
Experimental approach for assessing ion channel activity in the aorta under hypoxic conditions. This schematic illustrates the investigation of various ion channels involved in vascular responses to hypoxia, including voltage-dependent Ca^2+^ channels, receptor-operated Ca^2+^ channels, endothelium-dependent ion channels, and ATP-sensitive K^+^ (K_ATP_) channels.

The aorta is the largest artery in the body, and its functions are vital for blood circulation. *In vitro* studies on aortic tissues were conducted to investigate how oxidative stress and hypoxia influence vascular functions, particularly through the regulation of ion channels^[58–59]^. Hypoxia was induced using H_2_O_2_ treatment, glucose depletion in Krebs solution, or exposure to nitrogen (N_2_) gas to create oxygen-deprived conditions. These experiments primarily focus on potential-dependent ion channels, K_ATP_ channels, receptor-mediated ion channels, and endothelial-dependent ion channels to investigate their role in vascular dysfunction caused by ROS and RNS (***Fig. 8***). Specifically, (1) potential-dependent Ca^2+^ channels were studied to understand how hypoxia influences calcium influx and its downstream effects on vascular smooth muscle contraction (***Fig. 6***); (2) receptor-mediated Ca^2+^ channels were analyzed for their roles in hypoxia-induced vascular dysfunction, including the effect of ROS on receptor sensitivity and activity^[60]^; (3) K_ATP_ channels were studied for their roles in maintaining vascular tone under hypoxic and oxidative stress conditions; (4) endothelium-dependent ion channels were assessed for their contribution to vascular relaxation and nitric oxide (NO)-mediated signaling under hypoxia^[61]^; and (5) Na^+^/Ca^2+^ exchanger and Ca^2+^-ATPase ion channels were studied for their involvement in calcium homeostasis and how their activity is altered by hypoxia and oxidative stress.

Specific blockers were used to further elucidate the functions of these ion channels. The experimental setup included an isometric force transducer to measure the contractile responses of aortic tissues. Data were recorded and analyzed using GoLink software, providing insights into the role of ion channel dysfunction in hypoxia-induced vascular disorders^[62]^.

These studies not only shed light on the molecular mechanisms of hypoxia-induced vascular dysfunction but also identify potential therapeutic targets for conditions associated with oxidative stress and hypoxia.

### *In vivo* and *in silico* studies

To validate the findings of *in vitro* studies, *in vivo* experiments were performed on animal models. The experimental animals were divided into groups: control, hypoxia-induced, and hypoxia-induced with treatment. After a predetermined period, tissues such as the brain, liver, and heart were collected to analyze oxidative damage and assess the effectiveness of the administered treatments^[63]^.

**Figure 9:**
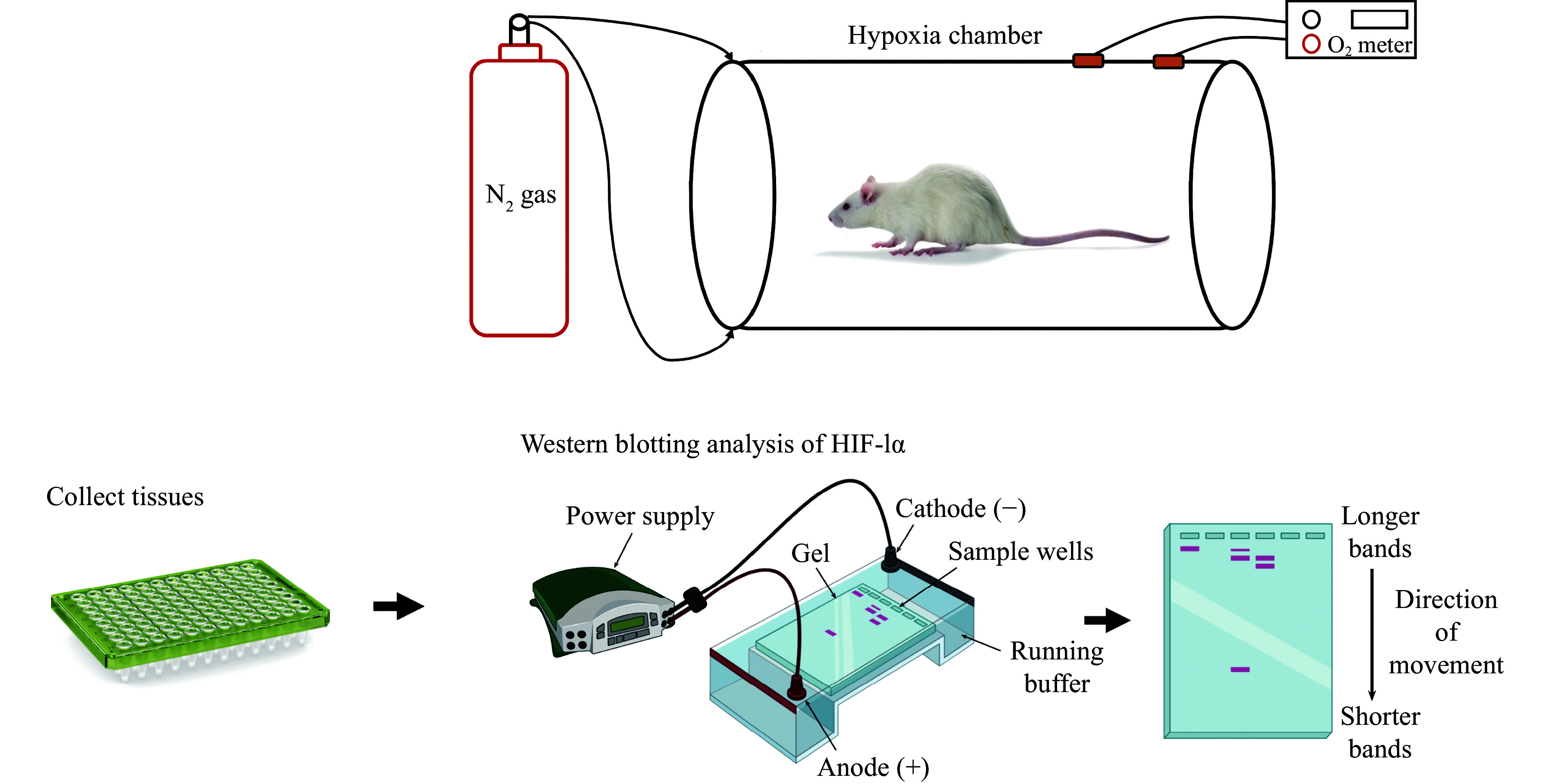
Experimental setup for inducing hypoxia in a rodent model and subsequent detection of hypoxia-inducible factor 1-alpha (HIF-1α) protein levels by Western blotting. The upper panel illustrates the hypoxia chamber system used for creating a controlled low-oxygen environment. Nitrogen gas (N_2_) is introduced into the sealed chamber to displace oxygen, and oxygen levels are continuously monitored using an O_2_ meter to ensure precise hypoxic conditions for the rodent. The lower panel shows the workflow for analyzing hypoxia-induced HIF-1α expression. After tissue collection from the treated animal, protein samples are prepared and loaded into wells of a polyacrylamide gel. Under an electric field, proteins migrate through the gel according to their molecular weight. Separated proteins are then transferred to a membrane and probed with specific antibodies against HIF-1α to visualize and assess the expression pattern, indicated by the appearance of specific bands on the blot.

One of the key techniques used in *in vivo* studies is Western blotting analysis, which quantifies HIF-1α levels in the cytosol (***Fig. 9***). HIF-1α is a critical marker of cellular adaptation to hypoxia and accumulates in cells under low-oxygen conditions. By comparing HIF-1α levels between treated and untreated groups, investigators can assess the efficacy of anti-hypoxic agents in preventing hypoxia-induced damage. The reduction in HIF-1α levels in treated animals serves as evidence that the treatment mitigates hypoxia and its harmful effects^[64]^.

*In silico* studies were used to model interactions between antioxidant compounds and cellular targets, such as ion channels and mitochondrial proteins, to predict efficacy and optimize drug design. These computational models provide a cost-effective approach to exploring potential mechanisms and identifying promising therapeutic candidates before proceeding to resource-intensive experimental studies.

### Treatment strategies for hypoxia-induced cardiovascular dysfunction

Antioxidants have been shown to prevent mitochondrial dysfunction and oxidative stress, which are key drivers of cardiovascular diseases like ischemia, hypertension, and myocardial infarction. By protecting mitochondrial ion channels, antioxidants help maintain calcium homeostasis, preventing excessive calcium influx that triggers cell death. Moreover, antioxidants preserve ATP production to meet cellular energy demands even under hypoxia^[65]^.

Therapeutic strategies focus not only on ROS neutralization but also on modulating key ion channels and enzymes, such as K_ATP_ and mPTP, which are vital in regulating cellular responses to oxidative stress^[66]^. The administration of antioxidants may modulate the activity of these channels, thereby preventing the cascade of events that lead to cellular dysfunction, apoptosis, and necrosis, which are hallmark processes in the progression of cardiovascular diseases^[67]^.

Through a combination of *in vitro*, *in vivo*, and *in silico* approaches, new antioxidant therapies may be developed to address the underlying causes of hypoxia-induced cardiovascular diseases. These interventions may reduce the global burden of cardiovascular conditions by preventing mitochondrial dysfunction, preserving energy metabolism, and maintaining cellular ion homeostasis in the face of oxidative stress.

#### Molecular dynamics in ischemia-induced hypoxia

In ischemia-induced hypoxia, the activation of HIF-1 plays a pivotal role in driving cellular adaptation to oxygen deprivation. Under normoxia, HIF-1 is tightly regulated by the von Hippel-Lindau protein (pVHL), an oxygen-sensitive E3 ubiquitin ligase^[68]^. pVHL recognizes and binds to hydroxylated proline residues on the HIF-1α subunit, targeting it for ubiquitination and subsequent proteasomal degradation. This mechanism ensures that HIF-1α levels remain low under normoxia, preventing the activation of hypoxia-responsive genes^[69]^. Under hypoxia, the activity of PHD, which hydroxylates HIF-1α, is inhibited because of the lack of oxygen as a substrate. As a result, pVHL cannot bind to HIF-1α, allowing it to stabilize and accumulate in the cytoplasm (***Fig. 2***). The stabilized HIF-1α then translocates to the nucleus, where it dimerizes with HIF-1β and binds to hypoxia response elements in the promoter regions of target genes^[70]^.

#### Pharmacological inhibition of pVHL

Blocking the activity of pVHL using specific inhibitors mimics hypoxic conditions by preventing the degradation of HIF-1α, even in the presence of oxygen. This pharmacological approach leads to the stabilization and activation of HIF-1α, promoting the expression of genes involved in hypoxia adaptation.

#### Gene expression and cellular adaptation

HIF-1 regulates the transcription of numerous genes critical for cellular survival under hypoxic conditions (***Fig. 3***). These include genes involved in angiogenesis (*e.g.*, *VEGF*) to improve oxygen delivery, glycolysis (*e.g.*, *GLUT1 *and *LDHA*) to enhance anaerobic ATP production, erythropoiesis (*e.g.*, *EPO*) to increase oxygen-carrying capacity, and cell survival and proliferation (*e.g.*, *BCL2* and *PDK1*) to mitigate hypoxia-induced cell death. By activating these pathways, HIF-1 facilitates cellular and tissue-level adaptation to hypoxia, reducing the detrimental effects of ischemia.

## Perspectives

Hypoxia-induced cardiovascular diseases present a complex interplay of molecular and cellular dysfunctions, with mitochondria at the center of these pathological processes. This review highlights the critical role of mitochondrial ion channels, ROS, and RNS in driving the detrimental effects of hypoxia on cardiovascular health.

Under hypoxia, mitochondrial dysfunction emerges as a pivotal contributor to cellular damage. The impairment of oxidative phosphorylation compromises ATP synthesis, disrupting the cellular energy balance. Dysregulation of mitochondrial ion channels, particularly the K_ATP_ and mPTP channels, exacerbates this dysfunction. The K_ATP_ channel, essential for maintaining the Δψ, becomes impaired, leading to excessive ROS release into the cytoplasm. Concurrently, the opening of the mPTP triggers apoptotic pathways, releasing pro-apoptotic factors such as cytochrome c and initiating cell death. These findings underscore the importance of targeting mitochondrial ion channels to mitigate hypoxia-induced cellular damage.

The role of ROS and RNS extends beyond oxidative damage, as these molecules interfere with critical signaling pathways. ROS disrupt lipid membranes and organelles (*e.g.*, the sarcoplasmic reticulum), while concurrently activating NOS, generating RNS to further damage cellular structures. In aortic smooth muscle cells, these actions result in ion channel dysregulation and abnormal relaxation, reducing vascular resistance and blood pressure. Although this adaptive response transiently lowers cardiac workload, the prolonged hypotension limits oxygen and nutrient delivery to vital organs, such as the brain, ultimately leading to ischemia and cell death. These insights provide a deeper understanding of how hypoxia-induced oxidative stress contributes to systemic cardiovascular dysfunction.

Therapeutic strategies targeting these pathways show substantial potential. Modulating the activity of mitochondrial ion channels, such as K_ATP_ and mPTP, may preserve the Δψm, reduce ROS production, and prevent apoptosis. Antioxidants, both natural and synthetic, have demonstrated efficacy in scavenging ROS and RNS, protecting cellular components from oxidative damage. Evaluating antioxidant properties using methods like DPPH, SOD, and MDA assays may aid in identifying potent therapeutic compounds.

Beyond mitochondria, restoring the functions of ion channels in aortic smooth muscle cells is crucial for maintaining vascular tone and preventing hypoxia-induced hypotension. Substances that regulate these ion channels under hypoxic conditions can modulate blood pressure and improve oxygen delivery to vital organs.

Furthermore, exploring molecular mechanisms through *in silico* and *in vivo* methods provides valuable insights for therapeutic development. Computational approaches targeting pVHL and PHD to inhibit their activity can enhance the stabilization and function of HIF-1α, a key regulator of cellular adaptation to hypoxia. Western blotting analysis of HIF-1α levels provides a reliable method for assessing the effect of therapeutic interventions on hypoxia-induced pathways.

## Conclusions

Chronic hypoxia is a pivotal factor in the progression of cardiovascular diseases, including ischemic heart disease, heart failure, and hypertension. The cellular response to prolonged oxygen deprivation triggers a cascade of molecular events, primarily driven by mitochondrial dysfunction, ROS generation, and disruption of calcium homeostasis. The opening of mPTP and dysfunction of mitoK_ATP_ channels further exacerbate these effects, leading to apoptosis and cell death. Oxidative stress, marked by excessive ROS and RNS, damages critical cellular structures, such as ion channels in the sarcoplasmic reticulum, thereby impairing cardiac muscle functions.

Understanding the complex pathways involved in hypoxia-induced damage, particularly mitochondrial pathways and oxidative stress, highlights the potential of antioxidants and ion channel modulators as therapeutic interventions. Targeting these molecular dysfunctions presents promising opportunities for developing treatments that significantly reduce the burden of cardiovascular diseases. This underscores the need for continued research using *in vitro*, *in vivo*, and *in silico* models to optimize these therapeutic strategies.

## References

[1] (2012). Cellular and molecular mechanisms of mitochondrial function. Best Pract Res Clin Endocrinol Metab.

[b2] 2Duchen MR. Mitochondria and calcium: from cell signalling to cell death[J]. J Physiol, 2000, 529(Pt 1): 57–68.

[3] (2022). Mitochondrial dysfunction: pathophysiology and mitochondria-targeted drug delivery approaches. Pharmaceutics.

[4] (2021). Mitochondria as a cellular hub in infection and inflammation. Int J Mol Sci.

[5] (2014). Mitochondrial biogenesis: a therapeutic target for neurodevelopmental disorders and neurodegenerative diseases. Curr Pharm Des.

[6] (2017). Mitochondrial function in hypoxic ischemic injury and influence of aging. Prog Neurobiol.

[7] (2020). Skeletal muscle energy metabolism during exercise. Nat Metab.

[8] (2024). The central role of the citric acid cycle in energy metabolism: from metabolic intermediates to regulatory mechanisms. Bioscience Evidence.

[9] (2025). Energy metabolism in health and diseases. Sig Transduct Target Ther.

[b10] 10Swarup S, Ahmed I, Grigorova Y, et al. Metabolic syndrome[M]. Treasure Island (FL): StatPearls Publishing, 2024.

[11] (2019). Shortage of cellular ATP as a cause of diseases and strategies to enhance ATP. Front Pharmacol.

[12] (2022). Mechanisms of ischemic heart injury. Cells.

[13] (2022). The burden of carbohydrates in health and disease. Nutrients.

[14] (2010). ATP synthase: from sequence to ring size to the P/O ratio. Proc Natl Acad Sci U S A.

[15] (2025). Acute CCl_4_-induced intoxication reduces complex I, but not complex Ⅱ-based mitochondrial bioenergetics—protective role of succinate. J Bioenerg Biomembr.

[16] (2001). Mitochondrial ATP-sensitive potassium channels inhibit apoptosis induced by oxidative stress in cardiac cells. Circ Res.

[17] (2011). Hypoxia. 4. Hypoxia and ion channel function. Am J Physiol Cell Physiol.

[b18] 18Baev AY, Abramov AY. Inorganic polyphosphate and F0F1-ATP synthase of mammalian mitochondria[M]//Müller WEG, Schröder HC, Suess P, et al. Inorganic Polyphosphates. Cham: Springer, 2022: 1–13.

[19] (2016). Endoplasmic reticulum stress and associated ROS. Int J Mol Sci.

[20] (2020). Bicalutamide elicits renal damage by causing mitochondrial dysfunction *via* ROS damage and upregulation of HIF-1. Int J Mol Sci.

[21] (2023). Endothelial cell dysfunction in cardiac disease: Driver or consequence?. Front Cell Dev Biol.

[22] (2022). Coronary blood flow in heart failure: Cause, consequence and bystander. Basic Res Cardiol.

[b23] 23Chaudhry R, Varacallo MA. Metabolic syndrome[M]. Treasure Island (FL): StatPearls Publishing, 2023.

[b24] 24Haddad A, Mohiuddin SS. Biochemistry, citric acid cycle[M]. Treasure Island (FL): StatPearls Publishing, 2023.

[25] (2022). Calcium overload and mitochondrial metabolism. Biomolecules.

[26] (2022). Physiological alterations of mitochondria under diabetes condition and its correction by polyphenol gossitan. J Microbiol Biotechnol Food Sci.

[27] (2025). Oxidative stress and inflammation in the pathogenesis of neurological disorders: Mechanisms and implications. Acta Pharm Sin B.

[28] (2024). The mitochondrial ATP-dependent potassium channel (mitoK_ATP_) controls skeletal muscle structure and function. Cell Death Dis.

[29] (2024). Unraveling the role and mechanism of mitochondria in postoperative cognitive dysfunction: A narrative review. J Neuroinflammation.

[30] (2024). Effect of polyphenols isolated from Plantago major L. and Plantago lanceolata L. on mitochondrial permeability transition pore in rat liver. Trends Sci.

[31] (2018). The role of KATP channels in cerebral ischemic stroke and diabetes. Acta Pharmacol Sin.

[32] (2003). Mitochondrial potassium transport: The role of the mitochondrial ATP-sensitive K+ channel in cardiac function and cardioprotection. Biochim Biophys Acta.

[33] (2018). Skeletal muscle fiber type in hypoxia: Adaptation to high-altitude exposure and under conditions of pathological hypoxia. Front Physiol.

[34] (2013). Neuroprotective role of ATP-sensitive potassium channels in cerebral ischemia. Acta Pharmacol Sin.

[35] (2002). Opening of mitochondrial K+ channels increases ischemic ATP levels by preventing hydrolysis. J Bioenerg Biomembr.

[36] (2015). Role of ATP-dependent K channels in the effects of erythropoietin in renal ischaemia injury. Indian J Med Res.

[37] (2013). Understanding metabolic regulation and its influence on cell physiology. Mol Cell.

[38] (2023). Oxidative stress contributes to inflammatory and cellular damage in systemic lupus erythematosus: Cellular markers and molecular mechanism. J Inflamm Res.

[b39] 39Alberts B, Johnson A, Lewis J, et al. Programmed cell death (apoptosis)[M]//Molecular biology of the cell. 4th edition. New York: Garland Science, 2002.

[40] (2015). Ion channels in the regulation of apoptosis. Biochim Biophys Acta (BBA)-Biomembr.

[41] (2023). A comprehensive review of natural products with anti-hypoxic activity. Chin J Nat Med.

[42] (2006). Coming up for air: HIF-1 and mitochondrial oxygen consumption. Cell Metab.

[43] (2007). Hypoxia-inducible factor (HIF)-1 regulatory pathway and its potential for therapeutic intervention in malignancy and ischemia. Yale J Biol Med.

[44] (2022). Correction of the mitochondrial NADH oxidase activity, peroxidation and phospholipid metabolism by haplogenin-7-glucoside in hypoxia and ischemia. Trends Sci.

[45] (2022). Vascular smooth muscle ion channels in essential hypertension. Front Physiol.

[46] (2024). *Inula helenium l*. root extract in sunflower oil: Determination of its content of water-soluble vitamins and immunity-promoting effect. Biomed Pharmacol J.

[47] (2013). Oxidative stress, mitochondrial damage and neurodegenerative diseases. Neural Regen Res.

[48] (2022). Mitochondrial calcium: Effects of its imbalance in disease. Antioxidants (Basel).

[49] (2025). *In vitro* and *in vivo* studies of *Crategus* and *Inula helenium* extracts: Their effects on rat blood pressure. Trends Sci.

[50] (2023). From imbalance to impairment: The central role of reactive oxygen species in oxidative stress-induced disorders and therapeutic exploration. Front Pharmacol.

[51] (2007). The role of reactive oxygen species and nitric oxide in mast cell-dependent inflammatory processes. Immunol Rev.

[52] (2021). The chemistry of reactive oxygen species (ROS) revisited: Outlining their role in biological macromolecules (DNA, lipids and proteins) and induced pathologies. Int J Mol Sci.

[53] (2015). Altered myocardial calcium cycling and energetics in heart failure—A rational approach for disease treatment. Cell Metab.

[54] (2008). Age-dependent effect of oxidative stress on cardiac sarcoplasmic reticulum vesicles. Physiol Res.

[55] (2004). Oxidative modification of rat cardiac mitochondrial membranes and myofibrils by hydroxyl radicals. Gen Physiol Biophys.

[56] (2023). Hypoxia-associated genes predicting future risk of myocardial infarction: a GEO database-based study. Front Cardiovasc Med.

[57] (2020). Mitochondrial electron transport chain: Oxidative phosphorylation, oxidant production, and methods of measurement. Redox Biol.

[58] (2024). Protective effect of DHQ-11 against hypoxia-induced vasorelaxation. Trends Sci.

[59] (2024). Targeting hypoxia signaling pathways in angiogenesis. Front Physiol.

[60] (2024). The protective effect of indole alkaloid vincanine against hypoxia-induced vasorelaxation model of rat aorta. Biomed Pharmacol J.

[61] (2012). Relaxant effect of the flavonoid pulicarin. Med Plant Res.

[62] (2024). The effect of Ajuga turkestanica on the rat aortic smooth muscle ion channels. Biomed Pharmacol J.

[63] (2014). Citric acid effects on brain and liver oxidative stress in lipopolysaccharide-treated mice. J Med Food.

[64] (1999). The tumour suppressor protein VHL targets hypoxia-inducible factors for oxygen-dependent proteolysis. Nature.

[65] (2023). Intracellular energy production and distribution in hypoxia. J Biol Chem.

[66] (2001). Targeting of HIF-α to the von Hippel-Lindau ubiquitylation complex by O_2_-regulated prolyl hydroxylation. Science.

[67] (2023). Reactive oxygen species, toxicity, oxidative stress, and antioxidants: Chronic diseases and aging. Arch Toxicol.

[68] (2018). Regulatory mechanisms of hypoxia-inducible factor 1 activity: Two decades of knowledge. Cancer Sci.

[69] (2024). Molecular exploration of natural and synthetic compounds databases for promising hypoxia inducible factor (HIF) Prolyl-4-hydroxylase domain (PHD) inhibitors using molecular simulation and free energy calculations. BMC Chem.

[70] (2022). Screening of prolyl hydroxylase 2 inhibitors based on quantitative strategy of peptides. J Chromatogr A.

